# Supplementary material for: Prevalence, intensity and risk factors of soil-transmitted helminthiasis after five effective rounds of preventive chemotherapy across three implementation units in Ondo State, Nigeria
Source: PLoS Negl Trop Dis. 2025 Jan 6;19(1):e0012533. doi: 10.1371/journal.pntd.0012533 (PMC11723620; doi:10.1371/journal.pntd.0012533)
Supplement: S1 File — (DOCX) [file pntd.0012533.s001.docx]

**Table A:** Sex and Age characteristics of study participants across the three LGAs.

|  |  | Ese-Odo | | | | Irele | | | | |  | Ile-Oluiji | | | |
| --- | --- | --- | --- | --- | --- | --- | --- | --- | --- | --- | --- | --- | --- | --- | --- |
|  |  | Sex (n, %) | | Age (n, %) | |  | Sex (n, %) | | Age (n, %) | |  | Sex (n, %) | | Age (n, %) | |
| SID | n | Female | Male | 5-9 | 10-14 | n | Female | Male | 5-9 | 10-14 | N | Female | Male | 5-9 | 10-14 |
| 1 | 50 | 25 (50.0) | 25 (50.0) | 22 (44.0) | 28 (56.0) | 49 | 22 (44.9) | 27 (55.1) | 18 (36.7) | 31 (63.3) | 24 | 10(41.7) | 14(58.3) | 19(79.2) | 5(20.8) |
| 2 | 49 | 20 (40.8) | 29 (59.2) | 18 (36.7) | 31 (63.3) | 48 | 22 (45.8) | 26 (54.2) | 20 (41.7) | 28 (58.3) | 35 | 14(40.0) | 21(60.0) | 17(48.6) | 18(51.4) |
| 3 | 48 | 23 (47.9) | 25 (52.1) | 20 (41.7) | 28 (58.3) | 50 | 32 (64.0) | 18 (36.0) | 20 (40.0) | 30 (60.0) | 31 | 22(71.0) | 9(29.0) | 15(48.4) | 16(51.6) |
| 4 | 44 | 17 (38.6) | 27 (61.4) | 22 (50.0) | 22 (50.0) | 51 | 25 (49.0) | 26 (51.0) | 22 (43.1) | 29 (56.9) | 50 | 22(44.0) | 28(56.0) | 18(36.0) | 32(64.0) |
| 5 | 42 | 21 (50.0) | 21 (50.0) | 12 (28.6) | 30 (71.4) | 50 | 25 (50.0) | 25 (50.0) | 28 (56.0) | 22 (44.0) | 44 | 23(52.3) | 21(47.7) | 16(36.4) | 28(63.6) |
| 6 | 50 | 22 (44.0) | 28 (56.0) | 20 (40.0) | 30 (60.0) | 50 | 32 (64.0) | 18 (36.0) | 17 (34.0) | 33 (66.0) | 21 | 7(33.3) | 14(66.7) | 4(19.0) | 17(81.0) |
| 7 | 50 | 26 (52.0) | 24 (48.0) | 20 (40.0) | 30 (60.0) | 59 | 29 (49.2) | 30 (50.8) | 26 (44.1) | 33 (55.9) | 51 | 25(49.0) | 26(51.0) | 28(54.9) | 23(45.1) |
| 8 | 50 | 27 (54.0) | 23 (46.0) | 31 (62.0) | 19 (38.0) | 51 | 25 (49.0) | 26 (51.0) | 21 (41.2) | 30 (58.8) | 50 | 28(56.0) | 22(44.0) | 29(58.0) | 21(42.0) |
| 9 | 50 | 23 (46.0) | 27 (54.0) | 22 (44.0) | 28 (56.0) | 49 | 24 (49.0) | 25 (51.0) | 20 (40.8) | 29 (59.2) | 37 | 19(51.4) | 18(48.6) | 18(48.6) | 19(51.4) |
| 10 | 51 | 25 (49.0) | 26 (51.0) | 24 (47.1) | 27 (52.9) | 48 | 24 (50.0) | 24 (50.0) | 18 (37.5) | 30 (62.5) | 43 | 18(41.9) | 25(58.1) | 22(51.2) | 21(48.8) |
| 11 | 49 | 23 (46.9) | 26 (53.1) | 26 (53.1) | 23 (46.9) | 50 | 25 (50.0) | 25 (50.0) | 20 (40.0) | 30 (60.0) | 50 | 24(48.0) | 26(52.0) | 17(34.0) | 33(66.0) |
| 12 | 50 | 25 (50.0) | 25 (50.0) | 25 (50.0) | 25 (50.0) | 50 | 25 (50.0) | 25 (50.0) | 22 (44.0) | 28 (56.0) | 37 | 17(45.9) | 20(54.1) | 21(56.8) | 16(43.2) |
| 13 | 48 | 18 (37.5) | 30 (62.5) | 15 (31.0) | 33 (68.8) | 51 | 22 (43.1) | 29 (56.9) | 21 (41.2) | 29 (58.0) | 49 | 22(44.9) | 27(55.1) | 19(38.6) | 30(61.2) |
| 14 | 43 | 16 (37.2) | 27 (62.8) | 19 (44.2) | 24(55.8) | 50 | 24 (48.0) | 26 (52.0) | 21 (42.0) | 29 (58.0) | 50 | 24(48.0) | 26(52.0) | 26(52.0) | 24(48.0) |
| 15 | 48 | 24 (50.0) | 24 (50.0) | 32 (66.7) | 16 (33.3) | 44 | 18 (40.9) | 26 (59.1) | 14 (31.8) | 30 (68.2) | 49 | 27(55.1) | 22(44.9) | 25(51.0) | 24(49.0) |
|  | 722 | 335(46.4) | 387(53.6) | 328(45.4) | 394(54.6) | 750 | 374(49.9) | 376 (50.1) | 308 (41.1) | 442 (58.9) | 621 | 302(48.6) | 319(51.4) | 294(47.3) | 327(52.7) |
|  |  | 0.90 |  | 0.014 |  |  | 0.67 |  | 0.87 |  |  | 0.48 |  | 0.001 |  |
|  |  |  |  |  |  |  |  |  |  |  |  |  |  |  |  |

SID: School ID

**Table B:** Soil-transmitted helminthiasis prevalence across the three LGAs.

|  |  | **Ese-Odo** | | | | **Irele** | | | | |  | **Ile-Oluiji** | | | |
| --- | --- | --- | --- | --- | --- | --- | --- | --- | --- | --- | --- | --- | --- | --- | --- |
| **SID** |  | **Asc** | **Hk** | **Tri** | **Any STH** |  | **Asc** | **Hk** | **Tri** | **Any STH** |  | **Asc** | **Hk** | **Tri** | **Any STH** |
|  | **n** | **n(%)** | **n(%)** | **n(%)** | **n(%)** | **n** | **n(%)** | **n(%)** | **n(%)** | **n(%)** | **n** | **n(%)** | **n(%)** | **n(%)** | **n(%)** |
| 1 | 50 | 29 (58.0) | 0 (0) | 2 (4.0) | 31 (62.0) | 49 | 5(10.2) | 0 (0) | 0 (0) | 5 (10.2) | 24 | 11(45.8) | 0 (0) | 0 (0) | 11(45.8) |
| 2 | 42 | 3 (7.1) | 0 (0) | 0 (0) | 3 (7.1) | 47 | 11 (23.4) | 5 (10.6) | 2 (4.3) | 13 (27.7) | 32 | 6(18.8) | 0 (0) | 0 (0) | 6(18.8) |
| 3 | 42 | 34 (81.0) | 0 (0) | 4 (9.5) | 34 (81.0) | 48 | 14 (29.2) | 0 (0) | 0 (0) | 14 (29.2) | 30 | 8(26.7) | 0 (0) | 0 (0) | 8(26.7) |
| 4 | 42 | 5 (11.9) | 0 (0) | 0 (0) | 5 (11.9) | 50 | 1 (2.0) | 0 (0) | 0 (0) | 1 (2.0) | 49 | 4(8.2) | 0 (0) | 0 (0) | 4(8.2) |
| 5 | 42 | 3 (7.1) | 0 (0) | 0 (0) | 3 (7.1) | 50 | 10 (20.0) | 0 (0) | 0 (0) | 10 (20.0) | 44 | 0(0) | 0 (0) | 0 (0) | 0(0) |
| 6 | 50 | 32 (64.0) | 0 (0) | 11 (22.0) | 32 (64.0) | 50 | 2 (4.0) | 0 (0) | 0 (0) | 2 (4.0) | 21 | 1(4.8) | 0 (0) | 0 (0) | 1(4.8) |
| 7 | 50 | 24 (48.0) | 0 (0) | 1 (2.0) | 24 (48.0) | 41 | 1 (2.4) | 0 (0) | 1 (2.4) | 1 (2.4) | 51 | 2(3.9) | 0 (0) | 0 (0) | 2(3.9) |
| 8 | 50 | 13 (26.0) | 0 (0) | 0 (0) | 13 (26.0) | 51 | 10 (19.6) | 0 (0) | 0 (0) | 10 (19.6) | 50 | 4(8.0) | 0 (0) | 0 (0) | 4(8.0) |
| 9 | 50 | 10 (20.0) | 0 (0) | 1 (2.0) | 10 (20.0) | 49 | 8 (16.3) | 0 (0) | 0 (0) | 8 (16.3) | 36 | 0(00 | 0 (0) | 0 (0) | 0(00 |
| 10 | 50 | 9(18.0) | 0 (0) | 0 (0) | 9 (18.0) | 48 | 1(2.1) | 0 (0) | 0 (0) | 1 (2.1) | 42 | 0(0) | 0 (0) | 0 (0) | 0(0) |
| 11 | 38 | 10 (26.3) | 0 (0) | 0 (0) | 10 (26.3) | 0 | 0(0) | 0 (0) | 0 (0) | 0(0) | 50 | 0(0) | 0 (0) | 0 (0) | 0(0) |
| 12 | 48 | 4 (8.3) | 0 (0) | 0 (0) | 4 (8.3) | 50 | 0(0) | 0 (0) | 0 (0) | 0(0) | 37 | 3(8.1) | 0 (0) | 0 (0) | 3(8.1) |
| 13 | 48 | 0(0) | 0 (0) | 0 (0) | 0 (0) | 46 | 0(0) | 0 (0) | 0 (0) | 0(0) | 49 | 0(0) | 0 (0) | 0 (0) | 0(0) |
| 14 | 41 | 0(0) | 0 (0) | 0 (0) | 0 (0) | 48 | 0(0) | 0 (0) | 0 (0) | 0(0) | 49 | 0(0) | 0 (0) | 0 (0) | 0(0) |
| 15 | 48 | 0(0) | 0 (0) | 0 (0) | 0 (0) | 41 | 0(0) | 0 (0) | 0 (0) | 0(0) | 49 | 0(0) | 0 (0) | 0 (0) | 0(0) |
|  | 691 | 176 (25.5) | 0 (0) | 19 (2.7) | 178 (25.8) | 668 | 63 (9.4) | 5 (0.7) | 3 (0.4) | 65 (9.7) | 613 | 39(6.4) | 0 (0) | 0 (0) | 39(6.4) |
|  |  | 0.001 | - | 0.001 | 0.001 |  | 0.001 | 0.001 | 0.064 | 0.001 |  | 0.001 | - | - | 0.001 |
|  |  |  |  |  |  |  |  |  |  |  |  |  |  |  |  |

SID: School ID; Asc: *Ascaris lumbricoides; Hk:* Hookworm; Tri: *Trichuris trichiura;*
