# Supplementary material for: Prevalence, intensity and risk factors of soil-transmitted helminthiasis after five effective rounds of preventive chemotherapy across three implementation units in Ondo State, Nigeria
Source: PLoS Negl Trop Dis. 2025 Jan 6;19(1):e0012533. doi: 10.1371/journal.pntd.0012533 (PMC11723620; doi:10.1371/journal.pntd.0012533)
Supplement: Attachment — Submitted filename: Response to Reviewer_R1.docx [file pntd.0012533.s002.docx]

**Response to Reviewers' Comments:**

| **Reviewer** | **Comments** | **Response** |
| --- | --- | --- |
| **Reviewer 1: General Comment** | Reviewer #1: Overall a well-written and clear study which fits well into the literature surrounding STH and prevalence monitoring. | *We thank the reviewer for their amazing contributions and review of the manuscript.* |
| **Reviewer 1: Specific comment** | **C1**: The objectives and testable hypotheses of this study are clear, the study is appropriately designed and follows equivalent methods used in other locations with a large enough sample size. Similarly, the sample population is well defined and mirrors similar studies in other locations. Statistical analyses are suitable and used well to support the conclusions. I have no concerns about the ethics of this paper. I am pleased to see that the funders was not involved with the study design or implementation as I was initially concerned that this would flag as a conflict of interest due to their involvement in the distribution of treatment. | ***Response 1****; We thank the reviewer for the positive feedback given on our manuscript. We have carefully revised the manuscript to address all other concerns raised. Please see below our responses to the comments.* |
|  | **C2:** I think some information about who the treated population were was overlooked. The author writes that the 'at-risk' population were administered with Albendazole but this group is not defined. This seems important as the author then later goes on to highlight the importance of reaching worm of reproductive age and it is unclear is this has been achieved here. | ***Response 2;*** *We thank the reviewer for this comment. We have revised the text, to highlight that albendazole was administered to children between ages 5 and 14 years. Please see Line 70* |
|  | ***C3:*** The analysis is well laid out and is clear to read, it follows the methods well. There are a few minor points: -• In line 55, I am unsure of the wording ‘marginally significant’ when referring to a p-value of 0.06. | ***Response 3;*** *This common phrase was used to communicate the almost significant p-value* |
|  | C4: -• In lines 46-47, it is unclear what the p-values are referring to and why there is no p-value stated for handwashing facilities. | ***Response 4;*** *We have now revised this text; the p-values show the proportion of access is significantly different across the LGAs. Please see the revised text.*  *“Access to improved sanitation (17.7%, 54.9%, and 58.2%), improved water sources (24.5%, 66.1%, and 69.8%), and handwashing facilities (9.0%, 39.6%, and 25.4%) were suboptimal and significantly varied across Ese-Odo, Irele, and Ile-Oluji, respectively (p < 0.05).”* |
|  | C5:-• On line 246-247 the language ‘significance level set at P<0.05’ is not statistically precise. It should read ‘significance level set at 0.05’ | ***Response 5;*** *We have revised this appropriately* |
|  | C6: -• Figure 1: The names of the regions are not easy to read | ***Response 6;*** *We thank the reviewers for this comment, we have provided a new figure, by increasing the font of the labels (also ensuring they do not cross the border lines). We have utilized the PACE tool from PLOS to ascertain its resolution.* |
|  | C7: -• Figure 2 & 3: There are schools outside of the region boundaries | ***Response 7;*** *We have corrected the maps. We thank the reviewer for this observation.* |
|  | C8: -• Table 3: It is unclear what the p-values are associated with | ***Response 8;*** *We agree with the reviewer. We have adjusted the presentation style by placing the p-value, on the columns rather than the rows. The p-value demonstrates if there were significant differences in the intensity categories observed across each LGA. Please see the new Table 3* |
|  | C9: -• Notation in tables is not consistent – sometimes n(%) is used, other times N(%) and in table 4, the numbers in the brackets are undefined and the reader assumes they are percentages. | ***Response 9;*** *We agree with the reviewer. We have revised all our tables. Table 1-7, ensuring the notations are consistent, and we also placed appropriate footnotes to explain any abbreviation or notation.* |
|  | C10:  The conclusions of this paper are written clearly and well supported by the data. some limitations are addressed but I have some concerns about unacknowledged biases: - • There is no mention of whether sampling government-owned primary schools introduces any bias. Are there non-government-owned primary schools? - • There is no mention of the bias introduced by the unsampled children. If they were unwell or absent from school, might this be due to a higher worm burden? | ***Response 10;*** *We thank the reviewer for this wonderful comment. We have included a section on limitations to capture the points raised by the reviewer. Please see lines 498-506*  *“Although this study demonstrates a significant reduction in worm burden across the study LGAs; however, we acknowledge potential bias due to the exclusion of non-government schools and non-enrolled children. Our sampling targeted government-owned primary schools, which serve most school-aged children in these areas, whereas non-government schools are few, sparsely populated, and generally have better WASH facilities. As a result, worm burden among children in public schools likely provides a more representative estimate of STH status. Additionally, while the absence of non-enrolled children and those unavailable during sampling may introduce some bias, our methodology adheres to WHO recommendations, supporting the validity of our findings despite these limitations”.* |
|  | C11:  The authors make the relevance and importance of this study very clear and discuss both why it is important in advancing the understanding and in addressing public health. | ***Response 11;*** *We thank the reviewer for this positive comment* |
|  | C12:  In addition to specific changes outline in the above sections, the following are small changes which may wish to be made. -• On line 103, the word ‘of’ is a typo | ***Response 12;*** *We thank the reviewer for this positive comment. We have revised the text accordingly.* |
|  | C13: -• On line 111 there is a missing word between ‘to’ and ‘decisions’ | ***Response 13;*** *We thank the reviewer for this positive comment. We have revised the text accordingly.* |
|  | C14: -• Species names not italicized | ***Response 14;*** *We thank the reviewer for this positive comment. We have revised the text accordingly.* |
|  | C15: -• The acronym SAC is defined more than once. | ***Response 15;*** *We thank the reviewer for this positive comment. We have revised the text accordingly.* |
|  | C16: -• Used R packages should be referenced in addition to the software. | ***Response 16;*** *We thank the reviewer for this positive comment. We have revised the text accordingly.* |
|  | C17: -• The terms ‘gender’ and ‘sex’ are used interchangeably but have different meanings. | ***Response 17;*** *We thank the reviewer for this positive comment. We have revised the text accordingly, replacing the word “gender” with “sex”* |
| **Reviewer #2: General Comment** | Reviewer #2: This study describes the effect of five or more effective rounds of albendazole PC for STHs in Nigeria. Biannual PCs were effective in an LGA with a baseline prevalence slightly above 50%, while annual PCs were not effective in an LGA with a 39% baseline prevalence and poorer WASH conditions. However, annual PCs were effective in an LGA with a 23% baseline prevalence and better WASH conditions. This study may support the adjustment of PC strategies based on WASH conditions. Additionally, it provides an update on the STH status in Ondo State, showing improvement compared to the ESPEN data from 2022. | *We thank the reviewer for their amazing contributions and review of the manuscript.* |
| **Reviewer # 2: Specific comments** | C1:  The design is appropriate to address the stated objectives. Sampling was reasonable, and the sample size is sufficient for impact assessment. However, some concerns remain: 1) The questionnaire used to evaluate individual MAM history was not sufficiently assessed. The data contains a history of PZQ exposure but lacks information on albendazole exposure. Including the information would provide a more comprehensive evaluation of the effect of risk factors. | ***Response 1;*** *We thank the reviewer for this positive comment on our study and sampling approach. We acknowledge the concerns raised by the reviewer, which is correct. As clearly mentioned by the reviewer, this study leveraged on a schistosomiasis impact assessment survey and there were concerns regarding the length of the questionnaire which limited the inclusion of several other questions. We have included this concern as part of the limitation of the study.* |
|  | C2: I am concerned about whether schools in Irele LGAs were selected after stratification by sub-districts. Some sampling sites appear to be clustered within a single district, which may affect the representativeness of the sample. | ***Response 2;*** *We thank the reviewer for this observation. Indeed, all schools were selected following stratification by sub-district, and we used proportional sampling allocations based on the population in each sub-district (i.e., the number of schools) to determine how many schools needed to be sampled. Specifically, we selected one primary sampling unit (PSU) for every five schools in an area. For example, sub-districts with 10 schools were allocated two schools randomly, while sub-districts with 20 schools had a larger allocation of five schools.*  *The sub-district in the map showing a concentration of schools is most urbanized, with a large population and a high density of schools along a central axis. Other areas are more forested and act as linkages to other sub-districts. Despite the clustering, we still observed a wide variation in endemicity within the sub-district, which may have been missed if we had sampled fewer schools.* |
|  | C3: According to Figures 2 and 3, some sampling sites seem to be located outside the boundaries of the LGAs. Please confirm whether these locations are correctly identified. | ***Response 3;*** *We have corrected the maps. We thank the reviewer for this observation.* |
|  | C4: It is recommended that the Kato-Katz (KK) method be performed on two slides per sample. While I understand the original intent was to evaluate schistosomiasis, using a single slide is less suitable for assessing STHs and their EPGs. | ***Response 4****; We agree with the reviewer that two smears are more suitable than one, although previous studies have reported high sensitivity from a single Kato-Katz smear. While we were constrained by resources for the project, we were only able to perform single smear and to improve the reliability of our findings despite using a single smear, we had two independent microscopists read and validate each slide for quality assurance.*  *We appreciate the reviewer's understanding of our situation. We have successfully leveraged on an assessment that was planned for schistosomiasis and have used this opportunity to advocate support towards impact assessment surveys that would help free up resources for acquiring more diagnostic commodities, which have been challenging to procure in our setting due to both cost and logistical constraints.* |
|  | C5: Lines 132–136: Please recheck the number of LGAs. It seems there are four LGAs, rather than three, with endemicity levels below 20%. | ***Response 5;*** *We thank the reviewer for this positive comment. We have revised the text accordingly.* |
|  | C6: The results are clearly presented, but some clarifications are needed: 1) If baseline data are available, the details should be provided. Rather than using the term "any STH," reporting the baseline prevalence for each specific STH will provide better insight into the baseline characteristics. If data on infection intensity at baseline are available, these should also be included. | ***Response 6;*** *We sincerely thank the reviewer for their insightful comment. In response, we have revised our manuscript, particularly Table 2 and the related text. We have provided baseline data at the species level for two of the three surveyed locations, as their surveys were conducted within 14 years. However, for the third location, the survey dates to 2008 (over 16 years), and species-level data were not traceable. Consequently, only the overall STH prevalence data, extracted from the country’s scorecard and program reports, could be included. Additionally, none of the available data reported intensity metrics, which we were therefore unable to incorporate.* |
|  | C7: As mentioned in the Methods section, the authors should provide the results of the stool examination for S. mansoni. A brief statement will suffice. | ***Response 7;*** *We thank the reviewer for this comment; However, we didn’t mention schistosomes or mansoni throughout the text of this manuscript (we did a word search to verify this). Our findings on schistosomiasis among these study populations have been published elsewhere.* |
|  | C8: The conclusions are supported by the data, but the limitations are not described. Since there are no baseline data for WASH, it seems difficult to distinguish the individual contributions of WASH improvements and PCs. | ***Response 8;*** *We appreciate the reviewer for this comment and we have now added a section describing the limitations of this study*  *Please see below*  *Although this study demonstrates a significant reduction in worm burden across the study LGAs; however, we acknowledge potential bias due to the exclusion of non-government schools and non-enrolled children. Our sampling targeted government-owned primary schools, which serve most school-aged children in these areas, whereas non-government schools are few, sparsely populated, and generally have better WASH facilities. As a result, worm burden among children in public schools likely provides a more representative estimate of STH status. Additionally, while the absence of non-enrolled children and those unavailable during sampling may introduce some bias, our methodology adheres to WHO recommendations, supporting the validity of our findings despite these limitations. Finally, the absence of baseline data for WASH across the study locations makes it difficult to distinguish the individual contributions of WASH improvements and MDA.* |

.
