# Supplementary material for: Prevalence, intensity and risk factors of soil-transmitted helminthiasis after five effective rounds of preventive chemotherapy across three implementation units in Ondo State, Nigeria
Source: PLoS Negl Trop Dis. 2025 Jan 6;19(1):e0012533. doi: 10.1371/journal.pntd.0012533 (PMC11723620; doi:10.1371/journal.pntd.0012533)
Supplement: Peer review history

# Peer review: Decision Letter 0

**DOI:** 10.1371/journal.pntd.0012533.r001

**Authors:** María Victoria Periago, Krystyna Cwiklinski

4 Nov 2024

PNTD-D-24-01310Prevalence, intensity and risk factors of soil-transmitted helminthiasis after five effective rounds of preventive chemotherapy across three implementation units in Ondo State, NigeriaPLOS Neglected Tropical Diseases Dear Dr. Ekpo, Thank you for submitting your manuscript to PLOS Neglected Tropical Diseases. After careful consideration, we feel that it has merit but does not fully meet PLOS Neglected Tropical Diseases's publication criteria as it currently stands. Therefore, we invite you to submit a revised version of the manuscript that addresses the points raised during the review process. Please submit your revised manuscript within 60 days Jan 03 2025 11:59PM. If you will need more time than this to complete your revisions, please reply to this message or contact the journal office at plosntds@plos.org. When you're ready to submit your revision, log on to https://www.editorialmanager.com/pntd/ and select the 'Submissions Needing Revision' folder to locate your manuscript file. Please include the following items when submitting your revised manuscript:* A rebuttal letter that responds to each point raised by the editor and reviewer(s). You should upload this letter as a separate file labeled 'Response to Reviewers '. This file does not need to include responses to any formatting updates and technical items listed in the 'Journal Requirements' section below.* A marked-up copy of your manuscript that highlights changes made to the original version. You should upload this as a separate file labeled 'Revised Manuscript with Track Changes '.* An unmarked version of your revised paper without tracked changes. You should upload this as a separate file labeled 'Manuscript '. If you would like to make changes to your financial disclosure, competing interests statement, or data availability statement, please make these updates within the submission form at the time of resubmission. Guidelines for resubmitting your figure files are available below the reviewer comments at the end of this letter. We look forward to receiving your revised manuscript. Kind regards, María Victoria PeriagoAcademic EditorPLOS Neglected Tropical Diseases Krystyna CwiklinskiSection EditorPLOS Neglected Tropical Diseases

Shaden Kamhawi

co-Editor-in-Chief

PLOS Neglected Tropical Diseases

orcid.org/0000-0003-4304-636XX

Paul Brindley

co-Editor-in-Chief

PLOS Neglected Tropical Diseases

orcid.org/0000-0003-1765-0002

 **Journal Requirements:** **Additional Editor Comments (if provided):** [Note: HTML markup is below. Please do not edit.] **Reviewers' Comments:** Reviewer's Responses to Questions

**Key Review Criteria Required for Acceptance?**

As you describe the new analyses required for acceptance, please consider the following:

**Methods**

-Are the objectives of the study clearly articulated with a clear testable hypothesis stated?

-Is the study design appropriate to address the stated objectives?

-Is the population clearly described and appropriate for the hypothesis being tested?

-Is the sample size sufficient to ensure adequate power to address the hypothesis being tested?

-Were correct statistical analysis used to support conclusions?

-Are there concerns about ethical or regulatory requirements being met?

Reviewer #1: The objectives and testable hypotheses of this study are clear, the study is appropriately designed and follows equivalent methods used in other locations with a large enough sample size. Similarly, the sample population is well defined and mirrors similar studies in other locations. Statistical analyses are suitable and used well to support the conclusions. I have no concerns about the ethics of this paper. I am pleased to see that the funders had no involvement with the study design or implementation as I was initially concerned that this would flag as a conflict of interest due to their involvement in the distribution of treatment.

I think some information about who the treated population were was overlooked. The author writes the that the 'at-risk' population were administered with Albendazole but this group is not defined. This seems important as the author then later goes on to highlight the importance of reaching worm of reproductive age and it is unclear is this has been achieved here.

Reviewer #2: The design is appropriate to address the stated objectives. Sampling was reasonable, and the sample size is sufficient for impact assessment. However, some concerns remain:

1) The questionnaire used to evaluate individual MAM history was not sufficiently assessed. The data contains a history of PZQ exposure but lacks information on albendazole exposure. Including the information would provide a more comprehensive evaluation of the effect of risk factors.

2) I am concerned about whether schools in Irele LGAs were selected after stratification by sub-districts. Some sampling sites appear to be clustered within a single district, which may affect the representativeness of the sample.

3) According to Figures 2 and 3, some sampling sites seem to be located outside the boundaries of the LGAs. Please confirm whether these locations are correctly identified.

4) It is recommended that the Kato-Katz (KK) method be performed on two slides per sample. While I understand the original intent was to evaluate schistosomiasis, using a single slide is less suitable for assessing STHs and their EPGs.

5) Lines 132–136: Please recheck the number of LGAs. It seems there are four LGAs, rather than three, with endemicity levels below 20%.

**********

**Results**

-Does the analysis presented match the analysis plan?

-Are the results clearly and completely presented?

-Are the figures (Tables, Images) of sufficient quality for clarity?

Reviewer #1: The analysis is well laid out and is clear to read, it follows the methods well.

There are a few minor points:

-• In line 55, I am unsure of the wording ‘marginally significant’ when referring to a p-value of 0.06.

-• In lines 46-47, it is unclear what the p-values are referring to and why there is no p-value stated for handwashing facilities.

-• On line 246-247 the language ‘significance level set at P<0.05’ is not statistically precise. It should read ‘significance level set at 0.05’.

-• Figure 1: The names of the regions are not easy to read

-• Figure 2 & 3: There are schools outside of the region boundaries

-• Table 3: It is unclear what the p-values are associated with

-• Notation in tables is not consistent – sometimes n(%) is used, other times N(%) and in table 4, the numbers in the brackets are undefined and the reader assumes they are percentages.

Reviewer #2: The results are clearly presented, but some clarifications are needed:

1) If baseline data are available, the details should be provided. Rather than using the term "any STH," reporting the baseline prevalence for each specific STH will provide better insight into the baseline characteristics. If data on infection intensity at baseline are available, these should also be included.

2) As mentioned in the Methods section, the authors should provide the results of the stool examination for S. mansoni. A brief statement will suffice.

**********

**Conclusions**

-Are the conclusions supported by the data presented?

-Are the limitations of analysis clearly described?

-Do the authors discuss how these data can be helpful to advance our understanding of the topic under study?

-Is public health relevance addressed?

Reviewer #1: The conclusions of this paper are written clearly and well supported by the data. Some limitations are addressed but I have some concerns about unacknowledged biases:

- • There is no mention of whether sampling government-owned primary schools introduces any bias. Are there non-government-owned primary schools?

- • There is no mention of the bias introduced by the unsampled children. If they were unwell or absent from school, might this be due to a higher worm burden?

The authors make the relevance and importance of this study very clear and discuss both why it is important in advancing the understanding and in addressing public health.

Reviewer #2: The conclusions are supported by the data, but the limitations are not described. Since there are no baseline data for WASH, it seems difficult to distinguish the individual contributions of WASH improvements and PCs.

**********

**Editorial and Data Presentation Modifications?**

Use this section for editorial suggestions as well as relatively minor modifications of existing data that would enhance clarity. If the only modifications needed are minor and/or editorial, you may wish to recommend “Minor Revision” or “Accept”.

Reviewer #1: In addition to specific changes outline in the above sections, the following are small changes which may wish to be made.

-• On line 103, the word ‘of’ is a typo

-• On line 111 there is a missing word between ‘to’ and ‘decisions’

-• Species names not italicised

-• The acronym SAC is defined more than once.

-• Used R packages should be referenced in addition to the software.

-• The terms ‘gender’ and ‘sex’ are used interchangeably but have different meanings.

Reviewer #2: (No Response)

**********

**Summary and General Comments**

Use this section to provide overall comments, discuss strengths/weaknesses of the study, novelty, significance, general execution and scholarship. You may also include additional comments for the author, including concerns about dual publication, research ethics, or publication ethics. If requesting major revision, please articulate the new experiments that are needed.

Reviewer #1: Overall a well written and clear study which fits well into the literature surrounding STH and prevalence monitoring.

Reviewer #2: This study describes the effect of five or more effective rounds of albendazole PC for STHs in Nigeria. Biannual PCs were effective in an LGA with a baseline prevalence slightly above 50%, while annual PCs were not effective in an LGA with a 39% baseline prevalence and poorer WASH conditions. However, annual PCs were effective in an LGA with a 23% baseline prevalence and better WASH conditions. This study may support the adjustment of PC strategies based on WASH conditions. Additionally, it provides an update on the STH status in Ondo State, showing improvement compared to the ESPEN data from 2022.

**********

PLOS authors have the option to publish the peer review history of their article (what does this mean? ). If published, this will include your full peer review and any attached files.

If you choose “no”, your identity will remain anonymous but your review may still be made public.

**Do you want your identity to be public for this peer review?** For information about this choice, including consent withdrawal, please see our Privacy Policy .

Reviewer #1: No

Reviewer #2: **Yes: ** Hyun Beom Song

[NOTE: If reviewer comments were submitted as an attachment file, they will be attached to this email and accessible via the submission site. Please log into your account, locate the manuscript record, and check for the action link "View Attachments". If this link does not appear, there are no attachment files.] **Figure resubmission:** While revising your submission, please upload your figure files to the Preflight Analysis and Conversion Engine (PACE) digital diagnostic tool, https://pacev2.apexcovantage.com/. PACE helps ensure that figures meet PLOS requirements. To use PACE, you must first register as a user. Registration is free. Then, login and navigate to the UPLOAD tab, where you will find detailed instructions on how to use the tool. If you encounter any issues or have any questions when using PACE, please email PLOS at figures@plos.org. Please note that Supporting Information files do not need this step. If there are other versions of figure files still present in your submission file inventory at resubmission, please replace them with the PACE-processed versions. **Reproducibility:** To enhance the reproducibility of your results, we recommend that authors of applicable studies deposit laboratory protocols in protocols.io, where a protocol can be assigned its own identifier (DOI) such that it can be cited independently in the future. Additionally, PLOS ONE offers an option to publish peer-reviewed clinical study protocols. Read more information on sharing protocols at https://plos.org/protocols?utm_medium=editorial-email&utm_source=authorletters&utm_campaign=protocols

# Author comment: Author response to Decision Letter 0

**DOI:** 10.1371/journal.pntd.0012533.r002

3 Dec 2024

Attachment Submitted filename: Response to Reviewer_R1.docx

# Editor report: Decision Letter 1

**DOI:** 10.1371/journal.pntd.0012533.r003

**Authors:** María Victoria Periago, Krystyna Cwiklinski

16 Dec 2024

Dear Prof. Ekpo,

We are pleased to inform you that your manuscript 'Prevalence, intensity and risk factors of soil-transmitted helminthiasis after five effective rounds of preventive chemotherapy across three implementation units in Ondo State, Nigeria' has been provisionally accepted for publication in PLOS Neglected Tropical Diseases.

Before your manuscript can be formally accepted you will need to complete some formatting changes, which you will receive in a follow up email. A member of our team will be in touch with a set of requests.

Please note that your manuscript will not be scheduled for publication until you have made the required changes, so a swift response is appreciated.

IMPORTANT: The editorial review process is now complete. PLOS will only permit corrections to spelling, formatting or significant scientific errors from this point onwards. Requests for major changes, or any which affect the scientific understanding of your work, will cause delays to the publication date of your manuscript.

Should you, your institution's press office or the journal office choose to press release your paper, you will automatically be opted out of early publication. We ask that you notify us now if you or your institution is planning to press release the article. All press must be co-ordinated with PLOS.

Thank you again for supporting Open Access publishing; we are looking forward to publishing your work in PLOS Neglected Tropical Diseases.

Best regards,

María Victoria Periago

Academic Editor

PLOS Neglected Tropical Diseases

Krystyna Cwiklinski

Section Editor

PLOS Neglected Tropical Diseases

Shaden Kamhawi

co-Editor-in-Chief

PLOS Neglected Tropical Diseases

orcid.org/0000-0003-4304-636XX

Paul Brindley

co-Editor-in-Chief

PLOS Neglected Tropical Diseases

orcid.org/0000-0003-1765-0002

***********************************************************

# Editor report: Acceptance letter

**DOI:** 10.1371/journal.pntd.0012533.r004

**Authors:** María Victoria Periago, Krystyna Cwiklinski

Dear Prof. Ekpo,

We are delighted to inform you that your manuscript, "Prevalence, intensity and risk factors of soil-transmitted helminthiasis after five effective rounds of preventive chemotherapy across three implementation units in Ondo State, Nigeria," has been formally accepted for publication in PLOS Neglected Tropical Diseases.

We have now passed your article onto the PLOS Production Department who will complete the rest of the publication process. All authors will receive a confirmation email upon publication.

The corresponding author will soon be receiving a typeset proof for review, to ensure errors have not been introduced during production. Please review the PDF proof of your manuscript carefully, as this is the last chance to correct any scientific or type-setting errors. Please note that major changes, or those which affect the scientific understanding of the work, will likely cause delays to the publication date of your manuscript. Note: Proofs for Front Matter articles (Editorial, Viewpoint, Symposium, Review, etc...) are generated on a different schedule and may not be made available as quickly.

Soon after your final files are uploaded, the early version of your manuscript will be published online unless you opted out of this process. The date of the early version will be your article's publication date. The final article will be published to the same URL, and all versions of the paper will be accessible to readers.

Thank you again for supporting open-access publishing; we are looking forward to publishing your work in PLOS Neglected Tropical Diseases.

Best regards,

Shaden Kamhawi

co-Editor-in-Chief

PLOS Neglected Tropical Diseases

Paul Brindley

co-Editor-in-Chief

PLOS Neglected Tropical Diseases
